# Marine Polyhydroxynaphthoquinone, Echinochrome A: Prevention of Atherosclerotic Inflammation and Probable Molecular Targets

**DOI:** 10.3390/jcm9051494

**Published:** 2020-05-15

**Authors:** Aleksandr A. Artyukov, Elena A. Zelepuga, Larisa N. Bogdanovich, Natalia M. Lupach, Vyacheslav L. Novikov, Tatyana A. Rutckova, Emma P. Kozlovskaya

**Affiliations:** 1 G.B. Elyakov Pacific Institute of Bioorganic Chemistry, Far Eastern Branch of the Russian Academy of Science, 159 Prospect 100-letiya Vladivostoka, Vladivostok 690022, Russia; artyukova@mail.ru (A.A.A.); zel@piboc.dvo.ru (E.A.Z.); novolga_05@mail.ru (V.L.N.); tanya1119@yandex.ru (T.A.R.); 2 Medical Association of the Far Eastern Branch of the Russian Academy of Sciences (FEB RAS MO), Kirov Str., 95, Vladivostok 690022, Russia; lnbogd@mail.ru; 3 Primorye Regional Clinical Hospital No. One (SHI), Aleutskaya Str., 57, Vladivostok, Primorsky Krai 690091, Russia; 19lnatasha80@gmail.com

**Keywords:** atherosclerosis, cardiovascular diseases (CVD), oxidative stress, polyhydroxy-1,4-naphthoquinones, echinochrome A (EchA), Histochrome, Thymarin, mechanism of action

## Abstract

The effect of low doses of echinochrome A (EchA), a natural polyhydroxy-1,4-naphthoquinone pigment from the sea urchin *Scaphechinus mirabilis*, has been studied in clinical trials, when it was used as an active substance of the drug Histochrome^®^ and biologically active supplement Thymarin. Several parameters of lipid metabolism, antioxidant status, and the state of the immune system were analyzed in patients with cardiovascular diseases (CVD), including contaminating atherosclerosis. It has been shown that EchA effectively normalizes lipid metabolism, recovers antioxidant status and reduces atherosclerotic inflammation, regardless of the method of these preparations’ administrations. Treatment of EchA has led to the stabilization of patients, improved function of the intracellular matrix and decreased epithelial dysfunction. The increased expression of surface human leukocyte antigen DR isotype (HLA-DR) receptors reflects the intensification of intercellular cooperation of immune cells, as well as an increase in the efficiency of processing and presentation of antigens, while the regulation of CD95 + expression levels suggests the stimulation of cell renewal processes. The immune system goes to a different level of functioning. Computer simulations suggest that EchA, with its aromatic structure of the naphthoquinone nucleus, may be a suitable ligand of the cytosolic aryl cell receptor, which affects the response of the immune system and causes the rapid expression of detoxification enzymes such as CYP and DT diaphorase, which play a protective role with CVD. Therefore, EchA possesses not only an antiradical effect and antioxidant activity, but is also a SOD3 mimetic, producing hydrogen peroxide and controlling the expression of cell enzymes through hypoxia-inducible factors (HIF), peroxisome proliferator-activated receptors (PPARs) and aryl hydrocarbon receptor (AhR).

## 1. Introduction

Atherosclerosis is an inflammatory disease affecting arterial blood vessels. This disease, characterized by the accumulation of lipids and fibrous elements in the subendothelial space, is one of the major causes of myocardial infarction and ischemic stroke [1,2,3]. There is no unified theory of the emergence of atherosclerosis as part of cardiovascular diseases (CVD) established yet. Several causes of the disease are discussed, for example, the autoimmune response [4,5,6,7,8,9,10,11,12,13,14] and primary dysfunction of the antioxidant system (superoxide model) [15,16,17,18,19,20,21,22,23,24].

In the past two decades, statins have been widely used as drugs in the treatment of atherosclerosis. The mechanism of action of statins is associated with the inhibition of the rate-limiting stage of cholesterol biosynthesis—transformation of β-hydroxy-β-methylglutaryl-CoA (HMG-CoA) into mevalonate catalyzed by HMG-CoA reductase. By blocking HMG-CoA reductase, statins prevent the formation of lipids, which controls the function of several intracellular proteins. By acting on a class II major histocompatibility complex (MHC) transactivator (CIITA), statins can interfere with the transcriptional induction of class II MHC molecules, which reduces immune activation in plaques [19].

Recent studies have established that another category of anti-atherosclerotic drugs, belonging to a group of nuclear transcription factors known as peroxisome proliferator-activated receptors (PPARs) proteins can inhibit T cells in vitro. Activators of both PPARα (drugs of the fibrate class) and PPARγ (drugs of the thiazolidinedione family) can reduce T-cell activation, as shown by a decrease in the production of IFNγ (interferon gamma), TNF (tumor necrosis factors) and IL-2 (interleukin-2) [25]. Therefore, activation of PPARα or PPARγ can also beneficially affect atherosclerosis, dulling adaptive and innate immune responses. Therefore, statins activate PPARα and thus, indirectly, control lipid and glucose metabolism, vascular inflammation and thrombosis, and NF-κB-dependent activation of smooth muscle cells and monocytes [26].

At present, vitamins of group K (plastoquinone, menaquinone, menadione) are also used for the treatment of atherosclerosis [27]. Some of them can accumulate in arterial vessels and enhance the biosynthesis of matrix Gla protein (MGP), which prevents vascular calcification [27,28,29]. Recent studies have confirmed the positive role of menaquinones in reducing the risk of developing coronary artery disease and reducing the degree of calcification of arteries [30].

Recently, the aryl hydrocarbon receptor (AhR), a nuclear regulator of xenobiotic metabolism and environmental responses, has become a new player in the pathogenesis and therapy of CVD [31]. AhR has various physiological effects, depending on where it is expressed. Although there has been progress in understanding AhR-related CVD, crosstalk between the cardiovascular system and the microenvironment remains uncertain; therefore, studies of new natural ligands of this receptor are of great interest.

**Figure 1:**
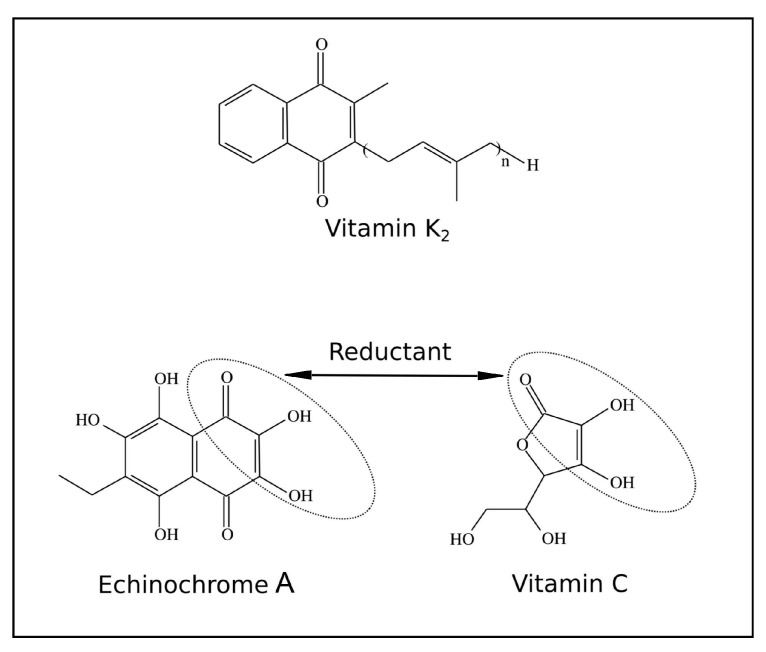
Structure of echinochrome A (EchA), K_2_ and C vitamins.

Herein, we report the results of treatment of patients having CVD, with the Russian medical drug Histochrome [32] and dietary supplement Thymarin [33,34,35], in both of which the natural pigment echinochrome A (EchA) is the active principle. EchA is 6-ethyl-2,3,5,7,8-pentahydroxy-1,4-naphthoquinone with the structural fragment similar to those of vitamins of K group and vitamin C (Figure 1). EchA enhanced mitochondrial biogenesis, increasing the mass of mitochondria and the functional effectiveness of oxidative phosphorylation (OXPHOS) in rat cardiomyocyte H9c2 cells [36,37,38]. Moreover, EchA treatment enhances transcription of mitochondrial genes, including PPAR-α, coactivator1 PPAR-γ (PCG-1 α), estrogen-bound α-receptor and nuclear respiratory factor-1 (Nrf-1), as well as transcription factors A, B2 and others [37,38,39].

Clinical studies of EchA in the composition of the drug and dietary supplements made it possible to evaluate its effect on homeostasis in patients with CVD and the possibility of its use in the treatment of CVD for the correction of metabolic, immunological and redox processes, as well as for prolongation and stabilization remission stage.

## 2. Experimental Section

### 2.1. Research Program

The legal basis for conducting clinical trials is the order of Roszdravnadzor dated June 18, 2009 No. 4818-Pr/09 to include the Medical Association of the Far Eastern Branch of the Russian Academy of Sciences (FEB RAS MO) and Primorye Regional Clinical Hospital No. One (SHI) in scroll health institutions with the right to carry out clinical research of medicines. The studies were conducted in agreement with the local ethics committee of FEB RAS MO (protocol No. 16 dated November 20, 2014) and SHI (protocol No. 18 dated October 20, 2014). The studies were conducted in a controlled environment of the hospital under supervision of qualified specialists (endocrinologist, therapist, neurologist, etc.). All persons involved in the conduct of clinical trials have professional education and experience, relevant tasks and certificates.

More than 140 patients with CVD were examined while studying the effect of EchA as part of the drug Histochrome and dietary supplement Thymarin. Medical control methods were used that characterized the clinical condition of patients. At the first visit, patients underwent a medical examination. Anthropometric indicators, a condition of the skin, digestive and cardiovascular systems (dynamics of blood pressure and pulse, electrocardiographic examination, Holter monitoring, ultrasound testing), systems of other organs and body systems and clinical and biochemical parameters of the blood were determined. Criteria for inclusion of patients in the study: patients with hypercholesterolemia, stable angina 3 functional class according to the Canadian classification, having ECG (electrocardiogram) criterion of a positive stress test. Criteria for exclusion of patients from the study: oncology and infectious diseases, diabetes mellitus, acute and chronic inflammatory diseases in the acute phase, cerebrovascular accident, renal and hepatic insufficiency syndromes and other severe concomitant diseases, congestive heart failure III and IV functional class according to NYHA (New York Heart Association), initial changes in ECG, which complicate its interpretation (left bundle branch block, pre-excitation syndrome) and renal and liver failure. Anthropometric and clinical characteristics, and risk factors of the study group, are shown in Table 1.

Using the adaptive randomization, 4 research groups were formed. The volume of research for each group did not differ. Patients gave written consent to participate in the study. Questionnaires were filled out for all patients, including assessment of complaints, history taking (including information on risk factors, among which smoking, arterial hypertension and burdened heredity for cardiovascular pathology were the most common), study of objective status and generally accepted studies (functional liver tests, blood glucose, coagulogram and lipid profile), ECG, stress test and coronary angiography in patients with stable exertional angina. In addition to traditional clinical, laboratory and instrumental studies, the research program included the study of oxidative and antioxidant status, endothelium nitroxide-producing function, determination of the concentration of the complex matrix metalloproteinase-9/tissue inhibitor of metalloproteinases-1 (MMP-9/TIMP-1) and determination of ischemic albumin. Peripheral blood sampling for biochemical studies was carried out from a cubital vein in an amount of 10 mL on an empty stomach from 8 to 9 a.m.; on the day of the study, smoking and nitrate intake were excluded, providing a 12-hour break. Plasma was obtained by centrifugation at t = +4 °C, at 3000 rpm for 20 min. The obtained samples were stored at t = −70 °C. The measurements were carried out in one series after collecting all samples. The patients were re-examined two weeks after the studies.

Patients with stable angina of functional class 3 received standard CVD therapies according to indications: a special diet, dosed exercise stress, lifestyle changes as well as the drug therapy—beta-blockers, inhibitors of the angiotensin-converting enzyme, antiplatelet agents, nitrates—upon request. The therapy already administered to the patients for the period of treatment with the preparation was not corrected.

The calculation of the sample size was carried out according to the formula:

N = 2 × (Za/2 + Zb) ^ 2/(d/SD) ^ 2,(1)

where N is the estimated sample size, Za/2 and Zb are the values of the normal distribution with probability a/2 and b, respectively, d is the clinically significant difference in group mean values and SD is the standard deviation. Sample size was calculated using the statmate software package.

The safety of EchA preparations was evaluated by detecting the symptoms identified as adverse events based on dynamic monitoring of the patient’s condition and monitoring of clinical and laboratory parameters.

### 2.2. Studies on the Clinical Effect of the Histochrome

The studies on the clinical effect of the preparation “Histochrome, Solution for Injections of 0.2 mg/mL in 1 mL Ampoules” (FSP R №002363/02-260213 Manufacturer’s Pharmacopeia Article) were conducted in FEB RAS MO. Using the adaptive randomization method, 2 groups were formed: the main group (*n* = 15: 13 women, 2 men) and the control-placebo group patients (*n* = 15: 13 women, 2 men), which included patients aged 53 to 70 with similar inclusion criteria (chronic ischemic мyocardial disease with comorbidities including atherosclerosis and hypertensive disease). All the female patients were in their post-menopausal period. One female patient of the main group dropped out of the study since in the course of the study a previously undetected cancer was diagnosed, and the patient was referred for treatment to the oncology center. We were interested in determining the reference range in order to assess the applicability of the used laboratory and instrumental methods. Therefore a control-volunteers group without chronic diseases was formed (*n* = 10: 8 women, 2 men). Patients of the main groups were registered at the therapist in the state of compensation and received standard therapy. Patients of the main and control-volunteers groups received Histochrome (EchA—0.2 mg, sodium carbonate—0.08 mg, sodium chloride from 0.9% to 1 mL) intramuscularly 2 mL per day for 10 days in day hospital, that is, 0.4 mg EchA per day (per course—4.0 mg). Patients of the control-placebo group received the same without EchA.

Drug tolerance was evaluated by subjective and objective signs, by clinical and laboratory indicators. Side effects associated with taking the drug were not observed. Marked changes in laboratory parameters (hematological, biochemical and hemostatic) systems were not detected. The average clinical and biochemical parameters of the blood of patients corresponded to the norms. In patients with normal glucose after taking the drug, its level did not change. Against the background of drug therapy, regardless of the route of administration, there was a subjective improvement in the well-being of patients.

### 2.3. Clinical Effect of Thymarin Dietary Supplement Studies

The dietary supplement Thymarin (Sanitary-Epidemiological Conclusion No. 77.99.03.935.Б.000138.06.04 dated 14.06.2004, TU 9350-064-02698170-2004) has the following composition: fructose—1.5%, acid ascorbinic—0.3%, EchA—0.025%. The studies were conducted in SHI. Using the adaptive randomization method, 3 groups were formed (placebo, main group and comparison) comparable by gender and age (n = 30: 16 women, 14 men each). The groups included patients aged 53 to 70 with stable angina pectoris 3 functional class with concomitant diseases, including hypercholesterolemia, atherosclerosis and hypertension. All the female patients were in their post-menopausal period. Patients of all groups received standard CVD therapy. Additionally, patients of the placebo group received syrup (fructose—1.5%, acid ascorbinic—0.3%) orally by 5 mL 2 times a day (0 mg of EchA per day) on an empty stomach for 3 weeks. Patients of the main group additionally received only Thymarin orally by 5 mL 2 times a day (2.5 mg of EchA per day) on an empty stomach for 3 weeks (52.5 mg per course). Patients of the comparison group additionally received only atorvastatin at 20 mg daily for 12 weeks (1680 mg per course). Tolerance to food additives was evaluated by subjective and objective signs, by clinical and laboratory indicators.

### 2.4. Hematological Studies

Blood for hematological studies was sampled with vacuum tubes containing EDTA, for biochemical—tubes without preservative— and for studies of the hemostatic parameters—tubes with the addition of 3.8% sodium citrate solution. Tubes of whole blood were centrifuged at 400 *g* for 20 min. Blood serum was used to determine the biochemical parameters. Complete blood count was conducted on the Abacus blood analyzer (Diatron, Wiener Neudorf, Austria).

#### 2.4.1. Biochemical Parameters of the Blood Studies 

Biochemical parameters of the blood were evaluated on BioChem Analette analyzer (High Technology Inc., North Attleboro, MA, USA), according to the instructions attached to the kit: total protein, bilirubin (BR), alanine aminotransferase (ALT), aspartate aminotransferase (AAT), creatinine, urea, lactate dehydrogenase, creatine phosphokinase, triglycerides, total cholesterol and cholesterol of clustering high- (HDL), low- (LDL) and very-low-density lipoproteins (VLDL). Atherogenic index of plasma (AIP) was calculated as follows:

AIP (Conv. units) = (total cholesterol − HDL)/HDL.(2)

#### 2.4.2. Method for the Determination of MDA in Red Blood Cells

The method was based on the formation of a colored complex in the interaction of malondialdehyde (MDA) with thiobarbituric acid (TBA). Blood sampling: 0.1 mL heparin (diluted 5000 units/mL 10 times) per 3 mL of venous blood. Red blood cells (0.5 mL) were washed three times with cooled isotonic solution and were hemolyzed by adding 2.5 mL of distilled water to the tube. The hemolysate (0.3 mL) and 10% phosphorotungstic acid (0.3 mL) were added to 2.4 mL of 1/12N H_2_SO_4_, mixed thoroughly and kept at 250 °C for 10 minutes. The mixture was centrifuged at 3000 rpm for 10 minutes, the supernatant was decanted, and the surface layer of the precipitate was carefully washed twice with 1 mL of H_2_0. Then, 3.0 mL of H_2_O was added to the sediment, the precipitate was carefully triturated with a rod, and 1.0 mL of 0.8% dilution TBA (8 mg per 1 mL of 0.5 CH_3_COOH and 0.5 H_2_O per 1 person) was poured. The closed tubes were placed for 1 hour in a water bath at 100 °C. After boiling, the tubes were cooled under a stream of cold H_2_O and were centrifuged at 3000 rpm for 15 minutes. The supernatant was transferred to clean, dry tubes, and the absorbance was determined on a Stat Farx photometer 1900 (Awareness Technology, Palm City, FL, USA) at a wavelength of 532 nm, against H_2_O.

In each sample, Hb was determined. To this end, 0.1 mL of H_2_O was added to 0.1 mL hemolysate obtained. The optical density was measured at a wavelength of 540 nm against H_2_O. Calculation:

CHb mg/mL = E_Hb_ × 32.75, (3)

where CHb is the hemoglobin concentration, E_Hb_ is the extinction at a wavelength of 540 nm, 32.75 extinction coefficient;

CMDA_Hb_ nmol/g = E_MDA_ / CHb × 1932, (4)

where CMDA_Hb_ is the MDA_Hb_ concentration, E_MDA_ is the extinction at a wavelength of 532 nm, 1932 extinction coefficient.

#### 2.4.3. Method for the Determination of TOA and TAA in Blood Serum

Total oxidant (TOA) and antioxidant activity (TAA) were determined by the degree of inhibition of ferrous-ascorbate-induced oxidation of Tween-80 to MDA.

TOA determination: reagents—1% Tween-80; 40% trichloroacetic acid (TCA) solution; 0.25% solution of TBA. In a 96-well plate, 100 μL of Tween-80 and 10 μL of blood serum were added to the test sample, and an appropriate volume of distilled water was added to the control sample. Then, the samples were incubated at a temperature of 40 °C for 48 hours. After that, 50 μL of TCA was added to each sample and left at room temperature for 60 minutes. Then, it was centrifuged at 8000 rpm for 15 minutes. In a second 96-well plate, 100 μL supernatant was mixed with 100 μL of TBA and incubated at 100 °C for 30 minutes. Samples were cooled and extinction was measured at 532 nm against the control (distilled water). The calculation was carried out according to the formula:

OOA (in %) = 100 − (Ec − Eo)/Ec × 100%(5)

where Eo and Ec are the extinctions of the experimental and control samples, respectively.

TAA determination: reagents—1% aqueous solution of Tween-80; 1.0 mM aqueous solution of ferrous sulfate (FeSO4); 10.0 mM aqueous solution of ascorbic acid; 40% TCA; 0.25% aqueous solution of TBA. As described above, 100 μL of Tween-80, 10 μL of iron sulfate solution, 10 μL of ascorbic acid solution, 10 μL of serum were added, and the corresponding amount of distilled water was added to the control sample. It was incubated for 48 hours at 40 °C, and then 50 μl of TCA was added and the samples were treated as described above. The calculation was carried out according to the formula:

TAA (in %) = (Eo − Ec)/Ec × 100%(6)

where Ec and Eo are the extinctions of the control and experimental samples, respectively.

#### 2.4.4. Nitric Oxide (NO) Determination

The total level NO metabolites was measured using the colorimetric method. The stable metabolites were determined according to the protocol approved by the company Biogenesis (Poole, UK). The following reagents from Sigma (St. Louis, MO, USA) were used: cadmium powder, 30% solution of ZnSO_4_ in water, chromogenic reagents No. 1 (1% sulfanilamide in 3N HC1) and No. 2 (0.1% *N*-(1-naphthyl) ethylenediamine hydrochloride in distilled water), nitrite standard (100 μM NaNO_2_ in water). Before use, cadmium (200 mg) was activated by removing the oxide film in 1 mL of 0.1 mM HCl in a separate dish, the acid was washed with three portions of distilled water, and then treated with 1 mL of 5% CuSO_4_ and washed again with three portions of distilled water. Serum samples (0.3 mL) were diluted with 0.6 mL of water; proteins were precipitated with 0.1 mL of 20% ZnSO_4_ for 15 minutes with shaking and then centrifuged at 6000 rpm for 5 minutes. Five milligrams of activated cadmium were added to microtubes (1.5 mL) with 0.5 mL of supernatant, shaken well and incubated on a shaker at room temperature for 12 hours. After that, the samples were centrifuged again, and the obtained supernatants were analyzed for the content of nitrite ion. Samples in duplicate of 100 μL were placed in the well of the plate, and reagents No. 1 and then No. 2 were successively added to each 50 μL. The specific optical density at 570 nm was measured in samples on the microplate spectrophotometer μQuant (BioTek, Winooski, VT, USA) using a reference wavelength of 750 nm. The concentration of nitrite ion in the sample was calculated according to a calibration curve (0–100 μM nitrite standard/mL).

#### 2.4.5. Determination of the Complex MMP-9/TIMP-1

The determination of the level of the MMP-9/TIMP-1 complex was carried out according to the protocol approved by R&D Systems (Minneapolis, MN, USA). Antibodies to MMP in working dilution (4 μg/mL) of 100 μL were applied to each well on a 96-well Costar immunological plate (Corning, NY, USA) in each well; after incubation for 12 hours at room temperature, the plate was washed 3 times with washing buffer (0.05% Tween-20 in PBS, pH 7.2) at 300 μL per well. The binding sites on the plate were blocked by adding 200 μL of blocking buffer solution (1% BSA, 5% sucrose in PBS). The plate was incubated for 2 hours at room temperature with stirring on a shaker and then washed as described above.

To obtain a calibration curve, 100 μL of solutions of standard antigen (AG) diluted in buffer solution (50 mM Tris, 10 mM CaCl_2_ 0.15 mM, 0.05% Brif 35, pH 7.5) was added to the first 2 rows. A calibration curve was constructed, starting with AG concentrations of 0-3000 pg/mL, with all samples in duplicate. In the remaining wells of the plate, test samples diluted 1:3 with dilution buffer were added and incubated for one hour at room temperature with stirring on a shaker, and then the tablet was washed, as indicated above.

One hundred microliters of a second anti-TIMP-1 (AT) labeled with biotin at a concentration of 50 ng/mL in a dilution buffer solution was added to each well. The microplate incubated for one hour at room temperature with stirring on a shaker and then was washed as described above.

One hundred microliters of conjugate of streptavidin with horseradish peroxidase in a dilution of 1:200 in a dilution buffer solution was added to each well. The plate was incubated for 1 hour at room temperature with stirring on a shaker, and then washing was repeated as indicated above.

The enzymatic activity of peroxidase in each well of the plate was determined by adding 100 μL of substrate (5 mg of o-phenyldiamine and 0.005 mL of 37% H_2_O_2_ to 12 mL of phosphate-citrate buffer solution, pH 5.0). The plate was incubated in the dark at room temperature, and the reaction was stopped by adding 0.05 mL of 5% sulfuric acid to each well of the plate.

Optical absorption was measured on a μQuant spectrophotometer (BioTek, Winooski, VT, USA) at a wavelength of 492 nm. The concentration of the MMP-9/TIMP-1 complex in the samples was determined by the calibration curve taking into account the dilution of the sample before analysis.

#### 2.4.6. Immunological Parameters Studies

Immunological parameters were evaluated in whole blood with the addition of EDTA, using diagnostic test systems and FACS Calibur cytoflow meter from Becton Dickinson (Franklin Lakes, NJ, USA). Immunophenotyping of lymphocytes was conducted by the following CD markers: CD3+, 4+, 8+, 16+, 19+, 25+, 95+ and human leukocyte antigen DR isotype (HLA-DR).

C-reactive protein was measured in blood serum by enzyme multiplied immunoassay (one-stage “sandwich” option) using the sets from Hema-Medika (Moscow, Russia). μQvant microplate photometer (BioTek, Winooski, VT, USA) was used to record the results. Cytokines and their receptors (IL-1β, IL-4, IL-6, IL-8, IL-10, TNF-α, IFN-γ, STNF-RII, sTNF-RII, sIL-1RII, sIL-6R) were measured by EMA method using Elx 808 IU microplate photometer (BioTek, Winooski, VT, USA) according to the Nova Tec (Dietzenbach, Germany) kits instructions.

#### 2.4.7. Indicators Characterizing Lipid Peroxidation (LPO) and the Mechanisms of Antioxidant Protection (AOP) Studies

Indicators characterizing LPO and the mechanisms of AOP were evaluated on the following parameters: total peroxidase activity (TPO) in activity units, total oxidant activity (TOA) in %, total antioxidant activity (TAA) in %, free radicals of blood serum (FR) in units, nitric oxide (NO) in blood serum in micromoles/L, malondialdehyde (MDA) in erythrocytes in moles/g Hb, MDA in blood plasma in micromoles/L, according to the techniques described previously [40].

#### 2.4.8. Data Analysis and Statistics

The credibility of differences of the indicators was evaluated using Statistical Package for the Social Sciences SPSS 11/0 (IBM Company, Armonk, NY, USA). application software on t-criterion. All results were expressed as the mean ± the standard deviation (SD). The difference between the two means (m± SD) was significant at *p* < 0.05.

### 2.5. Exercise Stress

Conducting load tests to patients was performed on a bicycle ergometer with increasing load with constant monitoring of heart rate and ECG and recording blood pressure at regular intervals. The test was performed in a sitting position on a Tuntiri Ergometer (Turku, Finland) with 12-lead ECG recording according to the WHO methodology until a submaximal heart rate was reached (75% from the maximum for the corresponding age) or other test termination criteria (angina pectoris, BP more than 230/130 mm Hg, appearance of threatening rhythm and conduction disturbances, reduction of blood pressure, fatigue, pain in the legs, refusing to continue the study, etc.). The survey was conducted in the morning on an empty stomach. If the patient was taking a beta-blocker, the drug was discontinued at least 24 hours before the study. The test began with a training session for 2 minutes followed by a period of exercise. The load started at 25 watts, increasing by 25 watts every 3 minutes. The total training time did not exceed 20 minutes. The ECG in 12 standard leads was recorded initially, at the end of each step of the load, at the height of the load and at the end of 3, 6, 9 minutes of rest, and blood pressure was measured at the same time. Positive test criteria were considered to be a segment of ST ischemic displacement up > 1 mm in any of the leads except V1-2, where 2 mm or more is considered a lift, or down from an isoelectric line > 1 mm and lasting 80 ms from point J, the slow slope of the segment ST at point J + 80ms > 2 mm (a rapid Kosovo-ascending decrease in ST is not ischemic).

The statistical significance of differences in bicycle ergometry before, during and after exercise was calculated using the Fisher angular transformation method and Student’s T-test. The calculations were performed using the program Statgraphics 16.1 (Statgraphics Technologies, Inc., Plains, VA, USA).

### 2.6. Molecular Modeling

#### 2.6.1. The HuAhR Homology Modeling

Because no experimental 3D structure of human AhR (huAhR) was available, to generate its homology models, the crystal structure of holo PAS B domain of the human hypoxia-inducible factor 2α (huHIF-2α), which shared 30% structural homology with huAhR, was applied as a template. The coordinates of 3H82 [41] were obtained as the X-ray holo structure of HIF-2α from the RCSB Protein Data Bank. The huAhR homology modeling was performed with program MODELLER 9.11 (San Francisco, CA, USA) through an interface in the UCSF Chimera program [42,43]. The structural defects and the atoms clash were adjusted with the Structure Preparation module in Molecular Operating Environment software package (ver. MOE 2016.0802 CCG) [44]. To optimize the structures after adding the hydrogen atoms using a Protonate 3D module, the energy minimization was performed with Amber12: Extended Hueckel Theory (EHT) force-field-treated protein and nucleic acids with AMBER parameters [45] and small molecules with 2D Extended Hueckel Theory (EHT) [46]. The best model structure was ranked on the basis of the lowest total potential energy. Geometric and stereochemical qualities of the models were estimated using Protein Geometry. Ramachandran plots of phi (φ) and psi (ψ) torsion angles for all of the residues and the atoms clash for each model were checked and equalized by energy minimization. The RMSD values for 104 Cα-atoms of huAhR models with those of the template were calculated by the Protein Superpose in MOE CCG. The corrected and refined structures were used for the further modeling analysis.

#### 2.6.2. Protein-Ligand Docking

To optimize electrical properties and a configuration of EchA molecule, a semiempirical quantum chemistry software package MOPAC2009 (Colorado Springs, CO, USA) and semiempirical PM6 method [47], integrated into package Molecular Docking Server [48], were used.

#### 2.6.3. Molecular Dynamics Simulation

Computations of molecular dynamics simulation for solvated protein-ligand complexes in an Amber12:EHT force field were performed under conditions of constant pressure, 300 K and pH 7.0 using MOE program [44] for 1 μs. Prior to MD simulations, the whole system was equilibrated to reduce initial bad contacts. Equilibration consisted in energy minimization of the initial side chain, position with fixed backbone atoms, followed by a minimization with restrained carbon alpha atoms and a short molecular dynamics (100 ps). Computer simulation and theoretical studies were performed using the cluster CCU “Far Eastern computing resource” IACP FEB RAS (https://cc.dvo.ru, Vladivostok, Russia).

## 3. Results

### 3.1. Effects of EchA from Its Use in the Forms of Histochrome and Thymarin on Correction of Lipid Metabolism Disorders and Antioxidant Status in Patients with CVD

#### 3.1.1. The Drug Histochrome 

The course of intramuscular injections with the Histochrome led to an improvement in well-being of 93.3% of patients with CVD: symptoms of exertional angina were significantly reduced, as were hypertensive encephalopathy and heart failure. Assessment of improvement was carried out according to the following parameters: subjective (all patients showed improvement in well-being) and objective (increase in exercise tolerance, correction of lipid metabolism disorders, decrease in the level of MMP-9/TIMP-1 complex, increase in the level of nitric oxide metabolites, improvement of the immune status, correction of endothelial dysfunction). All patients subjectively reported increased exercise tolerance. Adverse events associated with administration of the preparation have not been reported, as there were no significant changes in the electrocardiogram indicators. In comparison with the control group, all the blood tests of the main group patients before treatment showed the effects of oxidative stress consequences: an increase in TPO, TOA, the levels of FR, NO and MDA and a decrease in TAA (Table 2). Introduction of only 0.4 mg/day EchA in Histochrome form during the 10 days decreased the values of certain parameters in the main group patients, in that TOA and FR levels in blood tended sometimes to even lower values when compared with the control group. Increase of NO content in the blood of patients in the experimental group compared to the healthy people and CVD patients was apparently due to the increased activities of NO-synthases. Relatively small changes in some parameters of the main group of patients were probably associated with a small dose and short period of the introduction of the drug. Nevertheless, the results clearly showed antiradical, antioxidant and vasodilatory properties of Histochrome.

**Figure 2:**
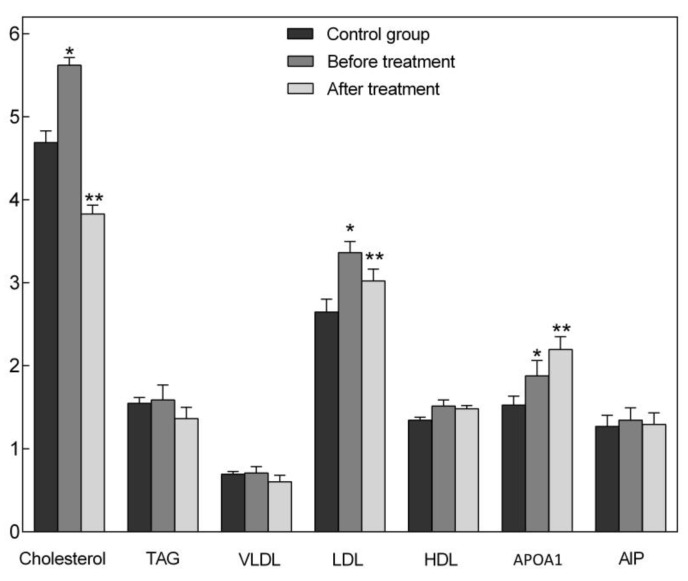
Indicators of lipid metabolism in patients of the control-volunteers and the main groups (before and after treatment with Histochrome, m ± SD-Standard Deviation). Abbreviations: Cholesterol: cholesterol, mmol/L; TAG: triglycerides, mmol/l; VLDL: cholesterol of very-low-density lipoproteins, mmol/L; LDL: cholesterol of low-density lipoproteins, mmol/L; HDL: cholesterol of high-density lipoproteins, mol/L; APOA1: apolipoprotein A1, g/L; AIP: (total cholesterol − HDL)/HDL. * differences with the control group were reliable at *p* < 0.05; ** differences before and after treatment were reliable at *p* < 0.05.

Changes in oxidative status of the blood also led to alterations in lipid metabolism. Effects of EchA in Histochrome form on the lipid metabolism in patients of the main group compared with those of the control group (healthy people) are shown in Figure 2. Lipid metabolism indicators of patients with CVD were higher than in healthy people (control group). After the treatment, there was a decrease in cholesterol, triglycerides (TAG), cholesterol of very-low-density lipoproteins (VLDL), cholesterol of low-density lipoproteins (LDL) and atherogenic index of plasma (AIP), when compared with the same patients before the treatment. However, these parameters did not reach the indicators of the control group. The level of apolipoprotein A1 (APOA1) was increased.

#### 3.1.2. Thymarin Dietary Supplement Effects

Three groups (placebo, main and comparison), with 30 patients in each with CVD, were formed to study Thymarin dietary supplement action. Hyperlipidemia (GL) of IIa type was diagnosed in 43% of patients and GL of IIb type- in 57% of patients. CVD diagnosis was confirmed clinically (stable angina 3 functional class without clinical manifestations of ischemic heart disease) data of echocardiography, bicycle ergometry and coronary angiography. Concomitant atherosclerosis was diagnosed by ultrasound imaging and the presence of free radical oxidation products in serum blood. Patients reported good tolerability to dietary supplements. No complications, allergic reactions or hypersensitivity was observed. As follows from Table 3, in the main group that received EchA in the form of the dietary supplement, the decrease in total cholesterol level was off 13.0% from the original, cholesterol of LDL, 17.3%; VLDL, 18.2%; TAG, 18.7%; and an increase in cholesterol of HDL was off 1.0%. The positive effect (some normalization of the lipid profile) was observed in 25 patients (83.3%), lack of the effect in 3 patients (10.0%) and the negative effect in 2 patients (6.7%). All patients of the main group noted an increase in performance, appearance of a sense of vivacity and improvement of the general mood and well-being, indicating sufficient energy supply of the body.

Analysis of the dynamics of the levels of TOA and TAA of the blood serum revealed that, in the case of administration of the dietary supplement Thymarin, patients in the main group had antioxidant status improved and the total content of pro-oxidants reduced. In patients treated with atorvastatin (comparison group), improvement of these indicators was not detected, which confirms the absence of its antioxidant properties. Due to the negative property of statins to increase the oxidative modification of LDL, the use of additional antioxidant therapy is a necessary measure.

Patient K (50 years old) was diagnosed with pathological liver steatohepatitis during initial testing, which is why he was not included in the experimental groups. He had suffered from hypertension for about 10 years with a maximum increase in blood pressure to 210/120 mm Hg, stable angina pectoris corresponding to 2 FC, about 2 years, type IIb GL. Prescribing statins is contraindicated in such patients. We decided to provide patient K with additional Thymarin therapy. The results are presented in Table 4.

Analysis of the dynamics of the lipid profile showed a decrease in total cholesterol by 24.1% from the initial level, LDL cholesterol by 43.6%, triglycerides by 31.8%, as well as the normalization of the atherogenic index. A positive effect was achieved in the state of oxidative status: the TOA of serum was reduced by 30%. In addition, the normalization of transaminases was indicative of a positive Thymarin effect on liver function. It is well known that atherosclerosis and hypercholesterolemia affect endothelium-dependent NO-stimulating regulation of vascular tone supported by nitric oxide generation [21,22]. When studying the effect of the dietary supplement, we have further examined the capability of EchA to correct endothelial dysfunction. The results of this study are given in Table 5.

As shown in Table 5, the additional administration of atorvastatin for 12 weeks or a dietary supplement for 3 weeks increased the level of NO metabolites.

In both groups of CVD patients, endothelial dysfunction was combined with an increase in content of metalloproteinase-9 (MPP-9) in the blood, and impairment of the function of extracellular matrix, as evidenced by the increase in the levels of MMP-9/TIMP-1 complex (TIMP-1 is a tissue inhibitor of metalloproteinase) to 5.64 ± 0.16 ng/mL and 5.12 ± 0.16 ng/mL, respectively, as compared to 2.77 ± 0.12 ng/mL in healthy persons. Administration of atorvastatin (for 12 weeks) or dietary supplement (for 3 weeks) resulted in a stabilization of the patient condition and improvement in the function of intracellular matrix. This was manifested by the reduction of the level of MMP-9/TIMP-1 complex to values of 4.10 ± 0.24 ng/mL and 4.77 ± 0.14 ng/mL, respectively, which was credibly different from the initially increased levels in the blood of patients (Table 4). As a result of a 3-week course of treatment of patients with hypercholesteremia (main group) by the dietary supplement, lipid metabolism was normalized and oxidative status indicators were improved, as well as nitro-producing endothelial function and functional activity of albumin. The level of MMP-9/TIMP-1 was decreased by 30% from initial values. Term of correction of lipid metabolism disorders using Thymarin (3 weeks) decreased by 2–4 times compared to 6–12 weeks when using atorvastatin. Considering that extracellular matrix dysfunction leads to a decrease in the elasticity of the vascular wall and a change in the response to the vasodilating effect of nitric oxide, the use of Thymarin helps to improve and, in some cases, restore the vasodilating function of the endothelium due to the improvement of matrix–cell interactions. Earlier studies [49] indicate that the use of Histochrome 1% significantly reduces the frequency and severity of clinical manifestations of coronary insufficiency in the form of angina in both acute and subacute myocardial infarction (MI), particularly with 10-day course of treatment. These clinical results allow us to recommend Thymarin for early prevention of atherosclerotic inflammation in patients with CVD along with statins. It should be noted that in the case of treatment with Thymarin, unlike statins, additional antioxidant therapy is not required. Thymarin does not exhibit the side effects typical of statins (muscle pain, fatigue, liver damage/transaminase, digestive upset, increased sugar, allergies, headache and dizziness). This was established on the basis of both subjective (indications of patients) and objective (stress test, biochemical analyzes).

### 3.2. Effect EchA on Hemostasis in CVD Patients

In the treatment of CVD patients, Histochrome and Thymarin virtually did not demonstrate significant effects on the basic values of their blood biochemical parameters (Table 6 and Table 7).

Increased levels of glucose circulating in the blood can cause microvascular damages that partly accelerate atherogenic processes due to the contribution to the disorder of endothelial dysfunction. We have studied the effect of low doses of EchA in the above-mentioned forms on carbohydrate metabolism of patients (Table 8). The obtained results (Table 6 and Table 7) suggest that EchA shows a regulatory action on the metabolism of glucose in patients.

Analysis of hematological indicators in patients of the main and the control groups did not reveal any changes beyond the physiological norm. But some differences between them have been detected (Table 9).

In the main group of patients, a slight decrease in mean corpuscular volume was observed, that, as well as having a tendency to a certain increase in hemoglobin level, was connected with an increase in its average concentration in erythrocytes (Table 9). After treatment with Histochrome, patients had a decreased total number of leukocytes, including lymphocytes (Table 9), which produce cytokines with pro- and anti-inflammatory activities. This indicated the reduction of inflammation and the action of EchA on immune-competent cells.

The analysis of hematological parameters, given in Table 9, shows a credible change in the number of lymphocytes in patients after treatment with Histochrome compared with those of the control and the main groups before treatment. In CVD patients of the main group, before treatment with the preparation, the number of lymphocytes in blood was increased in comparison with the control group. The drug treatment for 10 days partially decreased inflammation and reduced the total level of lymphocytes.

The changes in the cellular composition of lymphocytes were studied by using the immunophenotyping method (Table 10).

**Figure 3:**
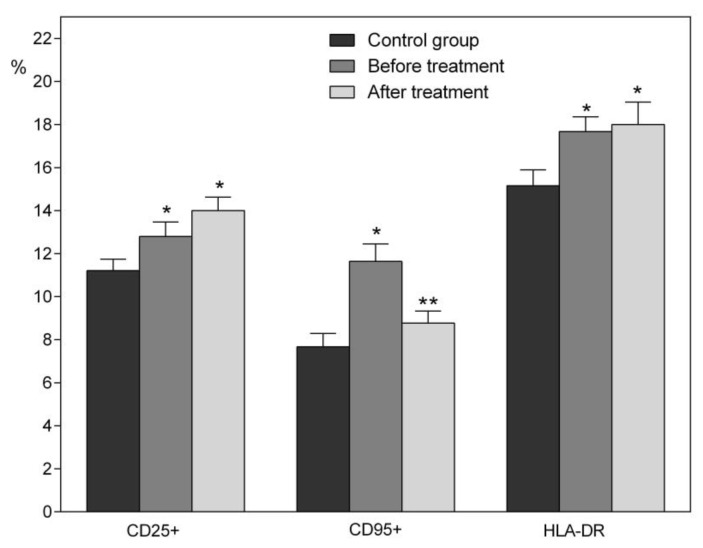
Relative content of lymphocytes expressing the activation and the differentiation markers in the blood in patients of the control-volunteers and experimental groups (before and after treatment with Histochrome, m ± SD). * differences with the control group were reliable at *p* < 0.05; ** differences before and after treatment were reliable at *p* < 0.05.

In patients of the main group before treatment, when compared with the control group, not only was an increase in the number of leukocytes observed, but quantitative changes also occurred in the composition of lymphocytes; for example, the number of T-cells (NKT, CD4^+^, CD8^+^) and B-lymphocytes (CD19^+^) were increased. The number of T-helper cells (CD3^+^, CD4^+^) remained virtually unchanged (Table 10). After the treatment with this drug, the patients had a decrease in the total number of leukocytes, T-helper cells and NKT-cells, and, on the contrary, the number of B-lymphocytes even increased. Patients with cardiac pathology had initially reduced immunoregulatory index (IRI) as a result of the preferential growth of CD8^+^ T-lymphocytes in comparison with a slight change in a T-helper cell subpopulation. The treatment with Histochrome resulted in a reduction of suppressor-cytotoxic subpopulation of IRI (Table 10). In patients of the main group, EchA induced changes to T-helper link and increased the number of B-lymphocytes (Table 10) and human leukocyte antigens (HLA-DR) (Figure 3). All these changes in the immune-competent cells probably indicate the strengthening of the humoral response to the atherosclerotic inflammation after treatment with Histochrome. It should be noted that the increase of the humoral link of immunity under the EchA effect was previously observed at the preparation of antisera against the influenza virus [50].

With atherosclerosis, the number of CD25+ receptors increases and does not reduce after the treatment of patients with the studied preparations (Figure 3). Table 11 summarizes the indicators of cytokine levels in patients of the control and main groups before and after treatment. Under the EchA action in patients with atherosclerosis, some changes in the composition of cytokines were found, which indicated a change in the ratio of Th1 and Th2 cells and in the heterogeneity of the macrophage population [51,52]. Usually, atherosclerotic plaque in atherosclerosis is dominated with Th1 [4,8,11]. As can be seen from the data given in Table 10, in test group patients after therapy with the preparation, there was a decrease in blood levels of not only pro-inflammatory cytokines IL-1β (producers: macrophages, monocytes and Th2), IL-6 (Th2), but also anti-inflammatory IL-10 (Th2 and T-reg) ones. At the same time, the number of pro-inflammatory IL-8, TNF-α (Th1) and INF-γ (Th1 and NKT) cytokines was slightly reduced. Particular attention shall be paid to the fact of the reduction in the number of IL-4 (Th2 and NKT) in the blood of patients after therapy with the preparation. Despite the increase in the number of CD25^+^ receptors that activate NKT cells, a slight increase in IL-4 in the blood of patients of the main group compared with the control is apparently associated only with this group of cells.

The immune system is regulated by distinct signaling pathways that control the development and function of the immune cells. An adaptive immune response is triggered via activation, differentiation and clonal expansion of the lymphoid lineage cells, T and B lymphocytes. It is now clear that Ahr regulates inflammatory signals via cross-talks with other signaling pathways such as the NF-B pathway. This pathway has long been known to control the expressions of IL-1, IL-6, IL-8, TNF- and other inflammatory genes.

The oxidative stress induced by AhR ligands may activate the noncanonical NF-κB and AP-1 pathways, resulting in an exacerbated inflammation [53]. Application of AhR agonists shifts the Th1/Th2 balance towardsTh1. We observed such a change in the immune status of the patients who suffered from atherosclerosis after the treatment with drugs based on EchA, whichis why it seemed to us to be of interest to start examining the capability of EchA to interact with the AhR.

### 3.3. Molecular Modeling of EchA-huAhR Interactions

AhR is a heterodimeric ligand-dependent receptor managed by structurally diverse exogenous and endogenous ligands (planar aromatic and hydrophobic compounds). It exhibits functional plasticity and causes a variety of biological responses, depending on ligands, that are associated with this receptor [54,55]. The anthropogenic AhR contains 848 amino acid residues forming three functional domains. The basic helix-loop-helix (bHLH) domain is located in the N-terminal region. This domain contains both nuclear localization (NLS) and nuclear export signal (NES) parts. Per-ARNT-Sim (PAS) domain including repeats PAS A and PAS B is believed to be responsible for protein–protein interactions upon dimerization and contains the binding sites for heat shock protein 90 (HSP90) chaperone and the transactivation domain (TAD). Moreover, PAS B region was indicated by the detailed mutagenesis and deletion experiments to be responsible for ligand binding (LBD) to the cytosolic AhR [56,57]. The C-terminal segment of AhR contains transcriptional activation domain (TAD). Recent extensive structural data on AhR:ARNT heterodimer structure and protein–DNA complexes of -bHLH-PAS systems have provided important information on how AhR:ARNT dimer specifically recognizes dioxin-response element [58]. At the same time, this structure lacks the PAS B domains. In this regard, a substantial interest represents understanding the molecular details of EchA interaction with AhR to realize its possible influence on the receptor activity. As the structure of huAhR ligand-binding domain (AhR LBD) has yet to be determined experimentally, a significant pool of structural information about the differences in interaction between the receptor and its various agonists, antagonists and selective modulators has been obtained from molecular simulation [55,59,60,61]. In this work, we focus our attention on understanding the molecular details of EchA interaction with huAhR by means of homology modeling, molecular docking and MD simulations.

At the first stage, the “agonist-optimized” homology model of the huAhR LBD was generated. Despite the low sequence similarity in the PAS domain containing-proteins, there is a high conservation of secondary structural elements (the α- and β-folds) within the family. Recent studies have shown that the holo huHIF-2α, considering the ligand-induced protein conformational rearrangements, represents a more suitable pattern for the construction of accurate homology models of avian, mouth and rat AhR LBDs than apo ones [41,60]. Based on these data, the 1.5 Å crystal structure of holo HIF-2α LBD with *N*-(furan-2-yl methyl)-2-nitro-4-(trifluoromethyl)aniline bound in the pocket (PDB ID 3H82, [41]) was chosen as a template to generate the huAhR homology model. The highest sequence similarity (31%) was identified for huAhR PAS-B domain with the human HIF-2α PAS-B, the 3D structure of which was experimentally elucidated. The model of huAhR spatial structure was generated with program MODELLER 9.11 (San Francisco, CA, USA) through an interface in the UCSF Chimera program (San Francisco, CA, USA) [42,43]. The Ramachandran Plot indicated the φ/ψ torsion angles within favorable and allowed regions for homology models. Residues with abnormal stereochemistry or the atom cloches were not observed. RMSD values of 104 huAhR models Cα-atoms with holo huHIF-2α ones were 0.75-0.86 Å for top-ranked models, testifying to high structural similarity of generated models to the template. In fact, the model structures showed a high conservation degree in β-sheet region and differed in the location of a few amino acid residues settled in the loop region. Three top-ranked homology models were then energy-minimized with MOE 2016.0802 CCG program and Ambar 12: EHT force field (San Francisco, CA, USA) [44,45] and selected for docking simulations.

**Figure 4:**
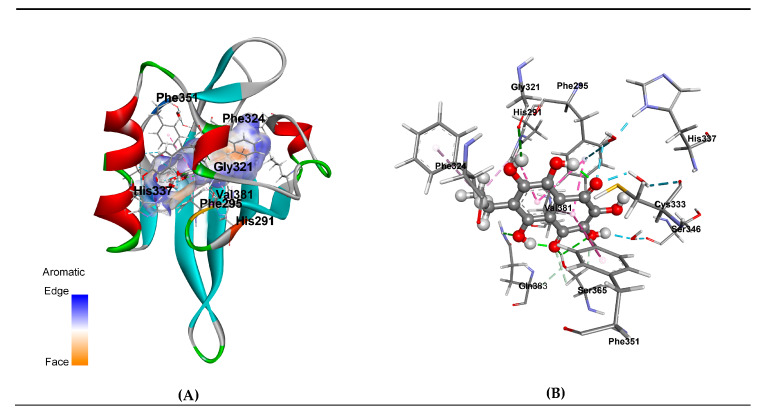
Spatial structural model of EchA complex with the ligand binding domain of human aryl hydrocarbon receptor (huAhR LBD). (**A**) Ribbon diagram of a structural model of EchA complex with huAhR LBD; EchA molecule is represented as ball and stick, colored according to elements; the hydrophobic cavity surface around the EchA is colored by aromaticity; residues involved in EchA binding represented as sticks and colored by elements. (**B**) A scheme of EchA intermolecular non-covalent interactions in complex with huAhR LBD; intermolecular non-covalent interactions are shown as dashed lines, and direct hydrogen bonds are colored in green; water-mediated hydrogen bonds in blue; π- alkyl, π-π stacked, and π-π T-shaped interactions in magenta.

Then, protein-ligand docking was performed without pre-known localization of EchA site (i.e., “blind” docking). Receptor models were kept rigid, and ligand was flexible and able to rotate the dihedral angles. As a result of “blind” molecular docking of EchA structure to the LBD of the receptor, the three-dimensional structure models of EchA-huAhR LBD complexes were generated. Our results showed no significant difference in EchA-receptor interaction mode for these three top-models and clearly demonstrated suitability of huAhR models to highlight the structural features of EchA interaction with huAhR. According to the architecture of the generated complex models, the EchA binding site is localized in the deep hydrophobic pocket of huAhR (Figure 4). EchA shares binding site with other well-characterized AhR ligands, such as the strongest agonists 2,3,7,8-tetrachlorodibenzo-p-dioxin (TCDD) and 6-formylindolo [3,2–b]carbazole (FICZ) as well as the synthetic AhR antagonist *N*-(2-(1H-indol-3yl)ethyl)-9-isopropyl-2-(5-methylpyridin-3-yl)-9*H*-purin-6-amine (GNF351) [62,63,64,65,66,67].

In addition, in order to understand the details of the interaction of the ligand with AhR, we performed molecular dynamics simulations of the EchA-huAhR complex in aqueous environment duration of 1 μs. Our simulation results revealed 19 receptor residues forming the EchA binding site (Table 12). Ten huAhR residues were involved in direct and/or water-mediated interactions with the EchA molecule and made a total contribution of -15.58 kcal/mol to the binding energy. Among these residues, there were Ser359, His285, Thr283, Phe289, Ile319, Phe345, Cys327, and Gln377, which have been earlier characterized as being involved in TCDD and other ligand/agonist binding on the basis of mutagenesis studies and functional analysis of mice AhR residues [65,66]. Taken together, these results have evidenced that AhR forms a stable enough complex with EchA. Furthermore, our computational data disclosed the hot-spot residues including Phe295, Phe351, His291, Ser365, Val381, and Cys333 to form the fingerprint of huAhR interaction with sea urchin pigment (with contribution to binding less than- 1.00 kcal/mol). Polar residues Ser365, Gln383, and Cys333 (corresponding to Ser359, Gln377, and Cys327 in mice AhR respectively) interacted with the ligand through direct and water-mediated hydrogen bonds. The contribution of these bonds to binding energy was estimated to be −2.300 kcal/mol, −0.446 kcal/mol and −1.170 kcal/mol, respectively. It is remarkable that sidechains of Phe295, Phe351, His291, Phe324 and Val381 are involved in π-π -Stacked, π-π-T-shaped and π-Alkyl interactions (Figure 4, Table 12) with the total estimated contribution of −9.637 kcal/mol that clearly indicate the crucial role of aromatic fragment of EchA structure in the influence on huAhR receptor activity and signal transduction.

## 4. Discussion

Being an antioxidant and a complexing agent, EchA chelates transition metal cations and thereby prevents the formation of the most aggressive and toxic ROS (reactive oxygen species). Once in the blood of patients, EchA is able to interact on the outer side of the plasma membrane of human cells not only with superoxide anion (O_2_·${}^{\bar{●}}$) produced by external cellular enzymes of the NOX and DUOX family, but also with oxygen (O_2_) [68,69,70,71]. As a result of this interaction, both types of oxygen turn into hydrogen peroxide (H_2_O_2_). Thus, EchA in reaction with a superoxide anion mimics the reaction of superoxide dismutase 3 (SOD 3) and protects human cells from the negative effects of the radical. Forming H_2_O_2_ from O_2_·${}^{\bar{●}}$ and O_2_, EchA involuntarily reduces the level of oxygen entering the cells, which immediately activates the work of HIF in the cells and allows cells to survive under conditions of hypoxia. In early studies of the pharmacological action of 1,4-naphthoquinones, the ability of these compounds to produce H_2_O_2_ and to lower the amount of reduced glutathione (GSH) was shown [72,73,74]. Later, we found that EchA reduces levels GSH (0.09 to 0.07 mm/L) and glucose in volunteers’ blood and also increases catalase activity (88.4% to 91.45%) during the first hour after oral EchA administration [35]. Three hours after EchA was taking, the level of catalase recovered to baseline (88.1%), and GSH even increased (0.11mm/L). EchA producing hydrogen peroxide in the body provokes the expression of PGC-1α co-activator of PPAR family receptors (H_2_O_2_ is the cause of overexpression of PGC-1α, the functioning of which increases the number of peroxisomes and mitochondria in the cells) [35,68,69,70,75,76,77]. The rising peroxisome number leads to an enhanced catalase content in the cell. At the same time, the decreased level of H_2_O_2_ in the cells exposed to catalase expressed in peroxisomes causes the lowering of CD95+ values after therapy of patients with Histochrome preparation (Figure 3). Therefore, H_2_O_2_ induces up-regulation of Fas (CD95+) in human endothelial cells [78]. The obtained data testified to the “oxidative stress” adaptive induction in the body of volunteers, caused by EchA. Experimental results demonstrating an increase in ATP (more than 80% compared to control) in the heart tissues of CVD patients who received EchA evidenced the importance of an intensification hexose monophosphate shunt by 1,4-naphthoquinones [49,79]. Those are EchA and its reduced form, which interact with oxygen and act on the same cell processes associated with accelerated glucose metabolism.

Hydrogen peroxide is capable of inducing the expression of Fas-ligand (CD178+) predominantly in activated cells of the immune system, such as Th1 and natural killers (NK) [80,81]. The simultaneous increase in the levels of both CD95+ in endothelial as well as other cells and CD178+ in immune cells (CD4+, CD8+) apparently contributes to the cell’s apoptosis. This is confirmed in our study by increased levels of creatine kinase MB—an intracellular marker of the cardiomyocytes decay in the blood of patients after treatment with EchA (Table 5). It should be noted that a delayed increase in T-lymphocytes (CD4^+^) was found in the blood of patients taking EchA [82]. Hydrogen peroxide and nitric oxide (NO), formed in endothelial and smooth muscle vascular cells after treatment with EchA, cause vasodilation and reduce ischemia and hypoxia [83].

Our data, summarized in Table 2, Table 3, Table 4, Table 5, Table 6, Table 7, Table 8, Table 9, Table 10 and Table 11 and Figure 2 and Figure 3, suggest that there is a transition of the immune system to a quite different level of functioning. The increased HLA-DR expression reflects the intensification of the immune cell intercellular cooperation as well as the increasing efficiency of antigen processing and presentation, while regulating of expression levels of the marker apoptosis CD95+ suggests the stimulations of cell renewal processes in the body.

The changes in cytokine profile observed in patients after their treatment with Histochrome indicate that EchA normalizes the population of macrophages, shifting their differentiation to M2 phase and thereby reducing the development of atherosclerotic inflammation. The anti-atherosclerotic effect of Histochrome is most likely associated with the activation of PPARs and nuclear factor Nrf2, the functioning of which is aimed at the urgent expression of such enzyme as DT-diaphorase, detoxifying compounds of quinonoid nature [35,78,81]. In addition to detoxifying functions, this enzyme plays an important protective role in CVD [84]. Apparently, EchA, being an agonist of PPARs, eliminates atherosclerotic inflammation by means of inhibiting of LDL oxidation, enhancement of the immune system functioning capacities and OXPHOS normalizing. Increased nitric oxide synthesis in vascular endothelial and smooth muscle cells in patients causes vasodilation and reduces ischemia and hypoxia.

Similar to bioflavonoids, EchA, having a planar skeleton of aromatic (aryl) hydrocarbon, can actively interact via its aryl moiety with a range of immunological cell receptors modulating their biological response and expression of various cytokines. Using computational methods, we also found that EchA binds to the aryl receptor (Figure 4), which may partly explain the observed effect of this naphthoquinone on the modulation of the immune response. Our computational study, including the homology modeling, molecular docking and MD simulation, revealed relatively small planar molecule of sea urchin pigment, EchA, owing to the aryl hydrocarbon structure, is retained strongly enough within AhR LBS hollow by stacking interactions with multiple intra-receptor exposed aromatic groups. Furthermore, the data have disclosed that among the residues responsible for close interaction with EchA, there are the ones defining the action of ligands not only as agonists but residues specific for antagonists binding solely, namely, Phe295, Phe324, and Gly321 (Table 11). The studies of site-directed mutagenesis have demonstrated overlapping amino acid residues within AhR LBD involved in the selectivity of the agonist or antagonist ligand binding mode and as a consequence of hsp90 binding, which mentions the ligand-binding conformation of AhR [62]. On the other hand, recently, A. Perkins and colleagues with the molecular simulation methods have identified the 307–329 residues as a flexible segment of the huAhR ligand pocket that adopts discrete conformations upon agonist or antagonist binding. This flexible segment of the AhR is proposed to be a structural switch that determines the agonist or antagonist activity of a given AhR ligand [64]. It should be particularly emphasized that the structure of 7-ethyl-2,3,5,6, 8-pentahydroxy-1,4-naphthoquinone (EchA) is the subject of special interest. The molecule of this compound is a highly oxidized derivative of the aromatic hydrocarbon naphthalene in which a benzene ring fused to a quinone ring. Protons of all hydroxyl groups are bound by intramolecular hydrogen bonds, among which the bonds between hydroxyl groups at positions 5 and 8 and carbonyl groups at positions 1 and 4 are very strong. These bonds form six-membered chelate cycles which can be considered as quasi-aromatic rings. As a result of the very fast transfer of hydrogen atoms in a quasi-aromatic cycle (so-called Enol–Enol tautomerism), the molecule of EchA can theoretically exist as an equilibrium mixture of two 1,4- and two 1,5-quinonoid forms. Quantum chemical calculations performed by the B3LYP density functional method in the 6-311G (d) basis set showed that 1,4-quinonoid tautomers are energetically more stable then 1,5-quinonoid ones [85]. Among 1,4-quinonoid tautomers, the form with ethyl group passed in a benzene ring is dominant. A close look at the structures of AhR ligands revealed that transformations of this kind are not characteristic for TCDD, FICZ, GNF351 and other AhR ligands (agonists or antagonists) [64], owing to the stability of these compounds’ molecular structures and the absence of any propensity to tautomeric conversions. Undoubtedly, the possibility of the change in the nature of aromatic rings in consequence of prototropic tautomerism is the unique feature of the EchA molecule [85]. This property enables EchA to slightly “drift” within the quite volumetric hydrophobic pocket between the polar side chains of Ser365, His291 residues of agonist-binding site and aromatic Phe295, Phe324 ones of antagonist binding site, possibly “switching” the receptor states, regulating gently the AhR activity and triggering a cascade of various cytokines expression. This result is in good agreement with the dynamics of cytokines profile as well as hematological parameters observed in the patients treated with Histochrome or Thymarin (Table 9, Table 11), and we can assume that homeostasis adjustment occurs as a result of the interaction of EchA with the aryl receptor.

It should be noted that over 400 genes are expressed by sea urchin *Strongylocentrotus purpuratus* in response to environmental stress [86,87]. Among them, there are the genes of transcription factors, in particular, homologs to aryl receptor, PPARs, HIF, nuclear factor Nrf2, heat shock proteins and nuclear hormone receptors that regulate the gene response to the stress in mammals [88,89]. EchA, being the main and unique metabolite of sea urchins, can apparently directly participate in the work of these metabolic regulators or control the consumption of various intracellular reactive types of oxygen, which leads to oxidative stress.

It should be emphasized that the whole-protein sequences of AhR of the sea urchin *S. purpuratus* and huAhR are identical within 27.28%, while the LBD of these receptors shares a higher sequence identity of 52.88%. This may indicate that mammalian receptors retain their specificity for the ligands from such marine sources as chordates and echinoderms. In humans, EchA, being an exogenous ligand, apparently also interacts with the aryl receptor and thereby initiates the expression of both P-450 cytochromes as well as DT-diaphorase and actively influences the change in homeostasis, including the immune system functioning capacity enhancement, and regulates circadian rhythms [30,88,89,90,91,92].

Thus, EchA not only has an antiradical effect and antioxidant activity but is also a SOD3 mimetic, producing hydrogen peroxide and controlling the expression of cell enzymes through HIF, PPARs and AhR. A decrease in the level of ROS in the body due to an increase in cellular and extracellular elements of antioxidant defense (antioxidant enzymes, mimetics of these enzymes and low molecular weight antioxidants) is the main key to the successful treatment of atherosclerosis [93].

The present study was limited by the criteria for inclusion and exclusion of patients in the study, a relatively small group of patients, fixed doses and the short duration of the experiment. The long-term effects of drugs have not been studied. The ethical limitations of the scientific research included inadmissibility of causing physical harm or harm to the honor and dignity of a person during an experiment; inadmissibility of involvement in the experiment or use of data about a person without obtaining his consent; limits, non-observance of which may result in irreversible changes in the biological (genetic) or conscious nature of a person. Despite the limitations, the study showed the promise of using EchA-based drugs in clinical medicine: they normalize lipid metabolism, but, unlike statins, they do not require additional antioxidant therapy and do not have their typical side effects (muscle pain, fatigue, liver damage (increased transaminase levels), digestive upset, increased sugar level, allergies, headache and dizziness). To determine the optimal dose, method and duration of administration of drugs based on EchA and the long-term effects of the drugs, it is necessary to conduct studies on a more representative group of patients.

## 5. Conclusions

Clinical studies of the effects of low doses of polyhydroxylated 1,4-naphthoquinone sea urchin in patients with CVD demonstrate the correction of lipid and carbohydrate metabolism disorders, as well as functional changes in the immune system. EchA, as a PPARs agonist, in addition to inhibiting LDL oxidation, moving the immune system to a higher level of functioning and normalizing OXPHOS, eliminates many causes of inflammation, exhibiting antioxidant and antiradical effects regardless of the method of its administration. The results of clinical studies allow us to offer drugs based on EchA as anti-inflammatory and anti-oxidant agents, and thus, they may be also beneficial for atherosclerosis and metabolic syndrome diseases. The identification of new molecular targets for EchA will expand the scope of applications of drugs based on it. In this study, we demonstrated for the first time that EchA has similar effects regardless of the route of administration. 

## Figures and Tables

**Table 1:** Anthropometric and clinical characteristics of the study group.

Total Surveyed	Anthropometric Characteristics			Risk Factors			Clinical Characteristics	
	Age	Men	Women	Smoking	Arterial Hypertension	A Burdened History of Cardiovascular Disease	Persons with Hyperchole- Sterolemia	Patients with Stable Angina 3 Functional Class
*n* (%)	year	*n* (%)		*n* (%)			*n* (%)	
140.0 (100)	53.63 ± 0.16	53 (38.86)	87 (62.14)	44 (31.42)	105 (95.24)	105 (95.24)	105 (95.24)	100 (71.14)

**Table 2:** Lipid peroxidation indicators in patients of the control and the main group (before and after treatment with Histochrome).

Group (*n*)	TPO (Units)	FR (Units)	NO (µmole/L)	MDA Erythro- Cytes (nmole/gHb)	MDA Plasma (µmole/L)	TAA (%)	TOA (%)
Control (*n* = 15)	449.0 ± 54.6	73.4 ± 3.4	13.9 ± 0.3	9.84 ± 0.31	3.54 ± 0.21	115.77 ± 1.85	13.49 ± 1.45
Before treatment (*n* = 15)	445.0 ± 60.0	72.8 ± 3.1	14.1 ± 0.4	10.50 ± 0.40	3.61 ± 0.22	110.0 ± 2.1	13.60 ± 0.87
After treatment (*n* = 15)	334.0 ± 26.0 *	67.2 ± 2.6 *	14.5 ± 0.7	10.17 ± 0.24 *	2.88 ± 0.23 *	110.0 ± 2.9	12.0 ± 0.58 *

* differences before and after treatment were reliable at *p* < 0.05. Abbreviations: TPO: Total peroxidase activity; FR: free radicals in blood serum; NO: nitric oxide; MDA: malondialdehyde; TAA: total antioxidant activity; TOA: total oxidant activity; *n*: sample size.

**Table 3:** Correction of lipid metabolism disorders and antioxidant status during treatment with Thymarin dietary supplement and atorvastatin in cardiovascular diseases (CVD) patients.

Indicators	Placebo Group		Main Group		Comparison Group	
	Before Treatment	After Treatment	Before Treatment with Dietary Supplements	After Treatment with Dietary Supplements	Before Treatment with Atorvastatin	After Treatment with Atorvastatin
	*n* = 30	*n* = 30	*n* = 30	*n* = 30	*n* = 30	*n* = 30
Total Cholesterol, mmol/L	5.46 ± 0.28	5.72 ± 0.47	6.21 ± 0.02	5.40 ± 0.13 *	6.49 ± 0.05	4.00 ± 0.24
Cholesterol of LDL, mmol/L	3.40 ± 0.32	3.90 ± 0.46	3.87 ± 0.16	3.20 ± 0.10 *	3.88 ± 0.21	2.43 ± 0.13
Cholesterol of HDL, mmol/L	1.71 ± 0.33	1.38 ± 0.12	1.30 ± 0.09	1.36 ± 0.06	0.99 ± 0.15	1.00 ± 0.10
Cholesterol of VLDL, mmol/L	0.78 ± 0.16	0.90 ± 0.12	1.10 ± 0.13	0.90 ± 0.07	1.12 ± 0.14	0.98 ± 0.08
Triglycerides, TAG, mmol/L	1.88 ± 0.29	1.73 ± 0.25	1.98 ± 0.15	1.61 ± 0.18	2.12 ± 0.20	1.88 ± 0.13
Total Antioxidant Activity, TAA, %	115.77 ± 1.85	115.82 ± 1.90	114.20 ± 1.05	121.10 ± 0.85 *	109.80 ± 2.50	109.70 ± 2.30
Total Oxidant Activity, TOA, %	13.49 ± 1.45	12.74 ± 1.34	14.50 ± 0.29	11.00 ± 0.32 *	14.60 ± 0.90	14.00 ± 0.30

* differences in indicators of patients’ performance before treatment and after treatment with the dietary supplement were reliable at *p* < 0.001. Abbreviations: *n*: sample size.

**Table 4:** The dynamics of some blood parameters in patient K.

Indicators	Before Treatment with Dietary Supplements	After Treatment with Dietary Supplements
Total cholesterol, mol/L	7.17	5.44
Cholesterol of LDL, mol/L	5.25	2.96
Cholesterol of HDL, mmol/L	1.19	1.15
Cholesterol of VLDL, mmol/L	0.73	1.33
Triglycerides, mmol/L	8.43	5.75
Atherogenic Index	5.03	3.07
Total Antioxidant Activity, TAA, %	119.00	124.00
Total Oxidant Activity, TOA, %	20.00	14.00
Aspartate Aminotransferase, AAT, U/L	87.00	43.00
Alanine Aminotransferase, ALT, U/L	68.00	27.00

**Table 5:** Correction of endothelial dysfunction in CVD patients.

Indicators	Control Group	Main Group		Comparison Group	
		Before Treatment with Dietary Supplements	After Treatment with Dietary Supplements	Before Treatment with Atorvastatin	After Treatment with Atorvastatin
	*n* = 30	*n* = 30	*n* = 30	*n* = 30	*n* = 30
Level of NO Metabolites, µmole/L	47.00 ± 0.43	39.94 ± 0.78 *	43.70 ± 0.82 *^,^ **	35.50 ± 0.65 *	40.10 ± 0.65 *^,^ **
Level of MMP-9/TIMP-1 Complex, ng/mL	2.77 ± 0.12	5.64 ± 0.16 *	4.10 ± 0.24 *^,^ **	5.12 ± 0.16 *	4.77 ± 0.14 *^,^ **

* differences in indicators of patients in the main and control groups were reliable at *p* < 0.001; ** differences in indicators of patients’ performance before treatment and after treatment with the dietary supplement were reliable at *p* < 0.001. Abbreviations: NO: nitric oxide; MMP-9/TIMP-1: the ratio of the concentrations of metalloproteinase-9 to tissue inhibitor of metalloproteinase-1; *n*: sample size.

**Table 6:** Indicators of biochemical blood tests in patients of the control and the main group (before and after treatment with Histochrome).

Group (*n*)	Total Protein (g/L)	Alb (%)	BR (µmole/L)	AAT (Units/L)	ALT (Units/L)	Creat (mmol/L)	Urea (mmol/L)	LDH (Units/L)	CPK (Units/L)	CPK MV (Units/L)	C-RP (mg/L)
Control (*n* = 15)	72.8 ± 0.8	51.4 ± 0.7	9.6 ± 0.2 *	28.2 ± 2.4	26.9 ± 3.6	76.2 ± 4.9	5.8 ± 0.5	214.3 ± 9.3	98.4 ± 8.7	20.5 ± 0.9	73.4 ± 0.6
Before Treatment (*n* = 15)	73.9 ± 0.7	52.1 ± 0.6	9.7 ± 0.1	28.6 ± 2.9	27.6 ± 4.6	75.0 ± 5.6	6.0 ± 0.3	230.2 ± 11.6	102.0 ± 9.2	20.8 ± 1.0	73.9 ± 0.7
After Treatment (*n* = 15)	75.2 ± 0.9	55.1 ± 0.8	8.6 ± 0.2 **	26.0 ± 1.5	23.5 ± 2.2	77.2 ± 3.1	6.3 ± 0.3	216.0 ± 12.7	102.0 ± 7.7	21.4 ± 1.2	75.2 ± 0.9

* differences with the group after treatment were reliable at *p* < 0.05. ** differences with the group before treatment were reliable at *p* < 0.05. Abbreviations: Alb: albumin; BR: bilirubin; AAT: aspartate aminotransferase; ALT: alanine aminotransferase; Create: creatinine; LDH: lactate dehydrogenase; CPK: phosphokinase, CPK MV: exit marker of creatine phosphokinase from cardiomyocites, C-RP: C- reactive protein; *n*: sample size.

**Table 7:** Indicators of biochemical blood tests in patients of the control and the main group (before and after treatment with Thymarin).

Group (*n*)	Total protein (g/L)	Alb (%)	BR (µmole/L)	AAT (Units/L)	ALT (Units/L)	Creatinine (mmol/L)	Urea (mmol/L)	Glucose (mmol/L)
Control (*n* = 15)	71.2 ± 0.7	43.7 ± 1.4	9.6 ± 0.20	28.2 ± 2.4	26.9 ± 3.6	76.2 ± 4.9	5.8 ± 0.5	5.9 ± 0.3
Before Treatment (*n* = 15)	72.6 ± 0.5	41.0 ± 1.4	8.90 ± 0.70	24.3 ± 1.2	24.0 ± 1.2	94.9 ± 3.3	5.7 ± 0.2	6.2 ± 0.4
After Treatment (n = 15)	73.1 ± 0.5	37.9 ± 0.7	8.40 ± 0.10	24.0 ± 2.0	24.0 ± 1.2	80.6 ± 3.0	5.6 ± 0.2	5.2 ± 0.2

Abbreviations: Alb: albumin; BR: bilirubin; AAT: aspartate aminotransferase; ALT: alanine aminotransferase; *n*: sample size.

**Table 8:** Indicators of carbohydrate metabolism in patients of the main group (before and after treatment with Histochrome).

Glucose(mmol/L) Before Treatment	Glucose(mmol/L) After Treatment	Lactate (mmol/L) Before Treatment	Lactate (mmol/L) After Treatment	C-peptide (ng/mL) Before Treatment	**C-peptide (ng/mL) After Treatment**
5.20 ± 0.08	5.06 ± 0.13	2.02 ± 0.18	1.79 ± 0.12	2.14 ± 0.27	2.43 ± 0.34

**Table 9:** Hematological parameters in patients of the control and the main group (before and after treatment with Histochrome).

Group (*n*)	Leucocytes (10^9^ cells/L)	Thrombocytes (10^9^ cells/L)	MCV (fl)	MCHC (g/L)	E %	CN %	S %	L %	M %
Control (*n* = 15)	7.1 ± 0.2	231.8 ± 14.8	82.4 ± 1.1	365.0 ± 2.6	2.4 ± 0.2	2.9 ± 0.2	53.6 ± 1.9	37.0 ± 1.8	5.0 ± 0.2
Before Treatment (*n* = 15)	7.2 ± 0.6	228.5 ± 12.9	81.8 ± 0.8	369.0 ± 2.7	2.0 ± 0.1	2.6 ± 0.1	52.3 ± 2.2	37.8 ± 1.9	4.8 ± 0.1
After Treatment (*n* = 15)	6.2 ± 0.3	299.0 ± 23.6 *	81.2 ± 0.8	370.8 ± 1.8	2.8 ± 0.1	3.3 ± 0.1	53.8 ± 1.6	35.0 ± 1.6	4.8 ± 0.1

* differences with the control group were reliable at *p* < 0.05. Abbreviations: MCV: mean corpuscular volume; MCH: mean concentration of hemoglobin in erythrocyte; E: eosinophils; CN: coli-nuclear neutrophils; S: segmented neutrophils; L: lymphocytes; M: monocytes; *n*: sample size.

**Table 10:** Indicators of the immune status in patients of the control and the main group (before and after treatment with Histochrome).

Group (*n*)	Leucocytes (10^9^ cells/L)	CD3^+^, CD4^+^		CD3^+^, CD8^+^		CD19+		IRI
		%	10^9^ cells/L	%	10^9^ cells/L	%	10^9^ cells/L	
Control (*n* = 15)	6.8 ± 0.4	47.10 ± 4.40	0.99 ± 0.01	27.00 ± 1.00	0.65 ± 0.04	11.50 ± 0.70	0.28 ± 0.02	1.7 ± 0.1
Before Treatment (*n* = 15)	7.2 ± 0.6	47.00 ± 4.11	1.00 ± 0.01	27.00 ± 1.70	0.70 ± 0.05	11.00 ± 0.60	0.30 ± 0.01	1.7 ± 0.1
After Treatment (*n* = 15)	6.7 ± 0.8	46.20 ± 0.02	0.90 ± 0.01	24.00 ± 1.00	0.50 ± 0.04 *	12.50 ± 0.60	0.20 ± 0.01	2.0 ± 0.1

* differences with the control group and before treatment were reliable *p* < 0.05. Abbreviations: IRI: Immunoregulatory index; *n*: sample size.

**Table 11:** Indicators of cytokines in the blood of the control and the main group patients (before and after treatment with Histochrome).

Group (*n*)	IL-1β (pg/mL)	IL-4 (pg/mL)	IL-6 (pg/mL)	IL-8 (pg/mL)	IL-10 (pg/mL)	TNFα (pg/mL)	IFNγ (pg/mL)	sTNF-RII (ng/mL)	sIL-1RII (ng/mL)	sIL-6R (ng/mL)
Control (*n* = 15)	25.6 ± 1.8	37.9 ± 2.7	35.0 ± 1.9	44.9 ± 3.8	42.8 ± 4.7	26.4 ± 2.4	60.2 ± 4.5	3.4 ± 0.3	7.9 ± 0.2	61.4 ± 3.4
Before Treatment (*n* = 15)	27.3 ± 1.3	38.9 ± 2.9	39.0 ± 3.9	45.7 ± 4.9	47.7 ± 9.3	26.6 ± 4.3	63.3 ± 14.1	3.5 ± 0.1	8.1 ± 1.2	60.2 ± 3.6
After Treatment (*n* = 15)	13.7 ± 2.9 *	27.6 ± 2.1	18.1 ± 2.9 *	39.4 ± 4.0	24.5 ± 4.5	22.1 ± 4.5	50.4 ± 4.3	3.6 ± 0.4	6.3 ± 1.3	55.0 ± 4.8

* differences with the control group and before treatment were reliable at *p* < 0.05. Abbreviations: IL: interleukin; TNFα: tumor necrosis factor; sTNF-RII, sIL-1RII, sIL-1RII, sIL-6R: soluble forms of receptors to the corresponding interleukins; *n*: sample size.

**Table 12:** Non-covalent interaction of EchA with huAhR LBD.

Type	Non-Covalent EchA Interaction with the Ligand Binding Domain of Human Aryl Hydrocarbon Receptor (huAhR LBD)	Agonist Binding Involved [64]	Antagonist Binding [64]
Hydrogen Bond	huAhR:Ser365:HG - EchA:O1	+	−
	huAhR:Ser365:HG - EchA:O2	+	−
	huAhR:Gln383:HE21 - EchA:O8	+	+
	EchA:H6 - huAhR:Gly321:O	−	+
	huAhR:Ser365:HB2 - EchA:O1	+	−
	huAhR:Ser365:HB3 - EchA:O2	+	-
Pi-Pi Stacked - Pi-Pi T-shaped	huAhR:Phe295 - EchA - huAhR:Phe351	up to 5%	+
	huAhR:Phe295 - EchA - huAhR:His291		
Pi-Alkyl	huAhR:His291 - EchA:C10	+	+
	huAhR:Phe324 - EchA:C10	−	+
	EchA - huAhR:Val381	+	up to 30%
Water-Mediated Hydrogen Bond	huAhR:His 337:HD - HOH - EchA:O4	+	+/−
	huAhR:Ser346:O - HOH - EchA:H2	+	-
	huAhR:Phe295: Pi-Orbitals - HOH - EchA:O4	up to 3%	+
	huAhR:Cys33:O - HOH - EchA:O4	up to 70%	up to 30%

“+” or “−” means that the residue is (or not) always involved in the interaction with a ligand of this type.
